# Depression score mediate the association between a body shape index and infertility in overweight and obesity females, NHANES 2013–2018

**DOI:** 10.1186/s12905-023-02622-7

**Published:** 2023-09-02

**Authors:** Qiangwei Pan, Xiaolu Shen, Hongfeng Li, Bo Zhu, Dake Chen, Jiajia Pan

**Affiliations:** 1 Department of Reproductive Endocrinology, Wenzhou People’s hospital, Wenzhou, 325000 China; 2 Department of Gynecology, Wenzhou People’s hospital, Wenzhou, 325000 China; 3 Department of Gynecology, the Affiliated People’s Hospital of Ningbo University, Ningbo, 315000 China; 4 Department of Urology Surgery, Wenzhou People’s hospital, Wenzhou, 325000 China

**Keywords:** ABSI, Infertility, Overweight, Obesity, Depression score, NHANES

## Abstract

**Background:**

Overweight and obese females demonstrate a significantly increased risk of anovulatory infertility. This study aims to investigate whether depression score could mediate the association between a body shape index (ABSI) and infertility, especially in overweight and obese population.

**Methods:**

We included 5431 adult female Americans from the National Health and Nutrition Examination Survey (NHANES, 2013–2018) database. ABSI manifested the body shape using waist circumference, weight, and height. Infertility or fertility status was defined by interviewing female participants aged ≥ 18 through the reproductive health questionnaires. Depression symptoms were assessed using the Patient Health Questionnaire-9 (PHQ-9) with total scores between 0 and 27. To investigate the association of infertility with ABSI and other individual components, survey-weighted multivariable logistic regression was performed. Mediation analysis of PHQ-9 score was conducted to disentangle the pathways that link ABSI to infertility among the NHANES participants.

**Results:**

596 (10.97%) females were categorized with having infertility among 5431 participants. Participants with infertility showed higher ABSI and PHQ-9 score, appearing greater population proportion with depression symptoms. In the multivariable logistic regression model, ABSI (adjusted odds ratio = 0.14, 95% CI: 0.04 to 0.50) and PHQ-9 (adjusted odds ratio = 1.04, 95% CI: 1.01 to 1.07) were positively associated with infertility. PHQ-9 score was estimated to mediate 0.2% (*P* = 0.03) of the link between ABSI and infertility in all individuals, but to mediate 13.5% (*P* < 0.01) of the ABSI-infertility association in overweight and obese adult females.

**Conclusion:**

The association between ABSI and infertility seems to be mediated by depression symptoms scored by PHQ-9, especially in those adult females with overweigh and obesity. Future studies should be implemented to further explore this mediator in ABSI-infertility link.

**Supplementary Information:**

The online version contains supplementary material available at 10.1186/s12905-023-02622-7.

## Introduction

Infertility, defined as the failure to achieve pregnancy after 12 months of sexual intercourse without any protection [1], affects approximately 14–25% of females all over the world and is often associated with significant physical and emotional stress [2]. Such a global health issue has attracted several researchers to figure out its etiology, however, there remains 15% of infertile couples have unexplained infertility [3].

Recently, some fundamental physical parameters have been reported to affect infertility, including excessive weight and waist circumference (WC). Females with a body mass index (BMI) greater than 27 demonstrate a significantly increased risk of anovulatory infertility compared with women with a normal-range BMI [4]. What’s more, female obesity (BMI > 30) is negatively associated with live birth rates following in vitro fertilization (IVF), drastically reducing the effectiveness of the treatment [5]. This is mediated by an interplay between derangements in the hypothalamic–pituitary–ovarian axis, oocyte quality and endometrial receptivity [6, 7]. Obesity has profound effects on sex hormone secretion and metabolism resulting in changes to the bioavailability of estrogen and androgen [8]. The overall adiposity is further associated with changes related to inflammation, coagulation, and fibrinolysis. Various such markers including C-reactive protein, IL-6, and TNF-α are found in increased levels in obese patients [9]. These have a deleterious effect on the reproductive cycle. The World Health Organization (WHO) has declared overweight and obesity rank fifth as worldwide death causes among risk factors [10], followed by high blood pressure, high blood glucose, and tobacco use. The prevalence of overweight and obesity are increasing among the adult population, which already exceeds 50%, especially in high- and middle-income countries [11]. Body mass index (BMI, calculated as weight in kilograms divided by height in meters squared) is limited in its ability to distinguish between muscle and fat accumulation, so doubt has been raised to its validity as an indicator of dangerous index [12, 13]. Waist circumference (WC) has emerged as a major complement to BMI. However, the high correlation of WC with BMI makes it hard to reflect body composition effectively.

Central adiposity strongly influences prevalence and clinical severity of female reproductive disorders [14, 15]. A body shape index (ABSI) has been recommended as a parameter that manifested the body shape using waist circumference, weight, and height [16]. which can better explain the degree of central abdominal adiposity [17, 18]. Nevertheless, the factors or pathways that mediate ABSI to infertility have remained unclear.

Psychological disorders are becoming more prevalent in modern society. Especially being obese significantly increases the likelihood of depression [19]. Scientists also have reported women with depressive symptoms tend to suffer from infertility, which could be a result of the altered secretion of sex cycle hormones and endometrial growth affected by emotional disorders [20, 21]. Depression, a highly prevalent mental disorder, is usually diagnosed by subjective self-reported questionnaires [22]. Patient health questionnaire-9 (PHQ-9), a self-administered version of a diagnostic tool for common mental diseases, was widely applied to depression detection and treatment [23]. People with a total PHQ-9 score over 10 were considered clinically depressed symptoms [24]. The important mediated role of depression disorder in quantities of diseases was reported, including inflammatory bowel diseases, breast cancer, and polycystic ovary syndrome (PCOS) [25–27].

This cross-sectional study included adult female Americans from the National Health and Nutrition Examination Survey (NHANES, 2013–2018) database. Associations of infertility with ABSI and other individual components were estimated using survey-weighted multivariable logistic regression. Besides, mediation analysis of PHQ-9 score was conducted to disentangle the pathways that link ABSI to infertility among the NHANES participants. Thus, the present study aims to investigate whether depression score could mediate the association between ABSI and infertility, especially in overweight and obese population.

## Materials and methods

### Data and participants

**Fig. 1:**
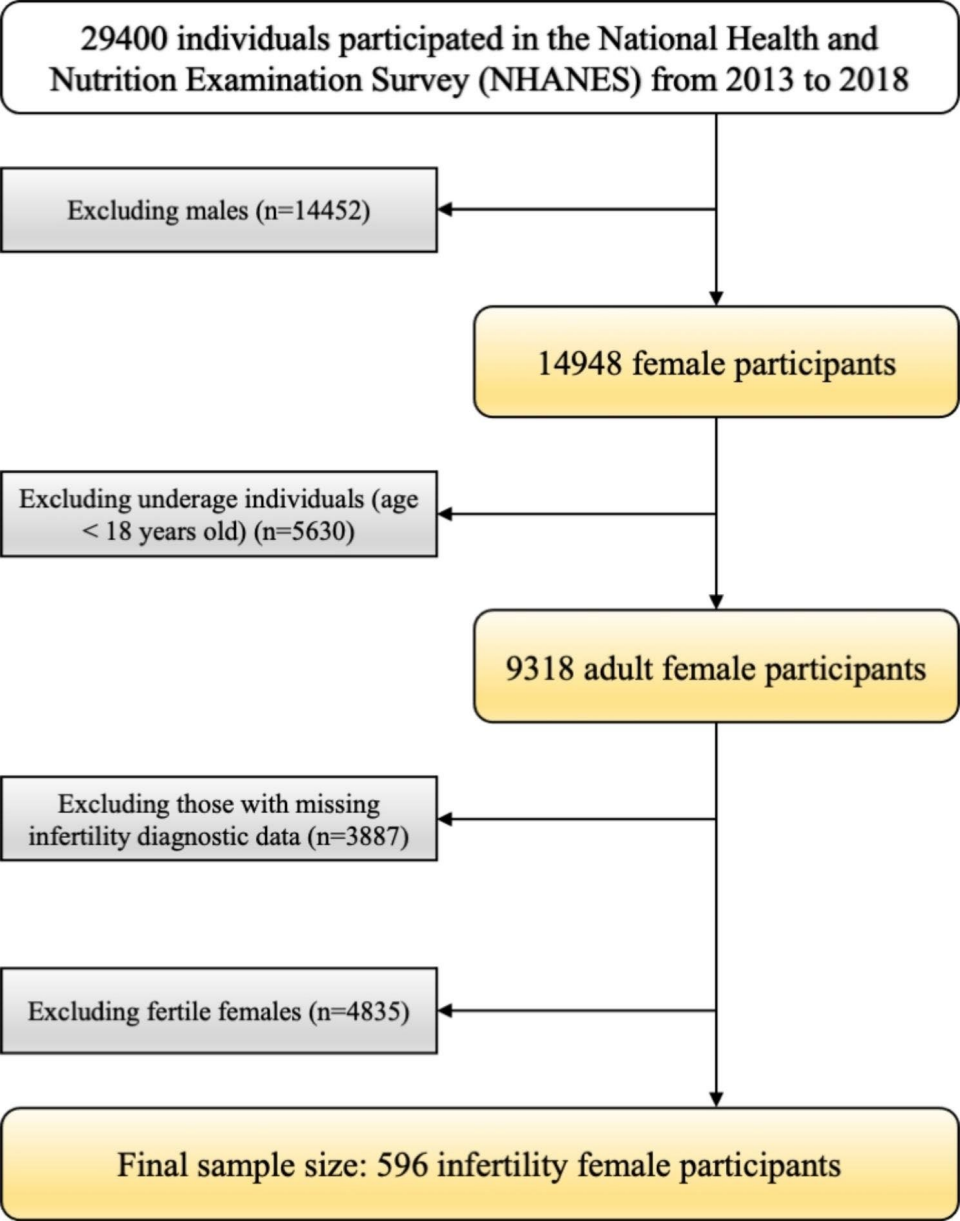
Flow chart demonstrating the subset of participants included for the following analysis from NHANES 2013–2018 dataset

Figure 1 demonstrated the inclusion criteria and workflow for this study. There was a total of 29,400 individuals who participated in NHANES from the 2013–2014, 2015–2016, and 2017–2018 year cycles. NHANES was a continuous cross-sectional study designed to estimate the health and nutritional status of adults and children in the United States over time. Each year, approximately 5000 people were surveyed as a nationally representative sample group. At least 10% of every interviewer’s work were randomly selected and validated by field supervisors via phone or field visit shortly after the completion of data collection. Data were collected from the three year-cycles, which included information concerning infertility in female patients. The sample size was estimated based on the criterion of Event Per Variable (EPV) [28]. Notably, EPV ≥ 10 was used to determine the minimal sample size required and the maximum number of candidate predictors that can be examined [29]. According to the study purpose, 14,452 males, 5630 underage females, and 3887 adult females without complete infertility diagnostic information were excluded. Ultimately, the study sample consisted of 5431 adult female participants whose infertility diagnostic data were available. Among them, 596 females were diagnosed as infertility.

### Infertility data

Infertility status was the outcome variable of interest. All female participants ages 18 or over were surveyed in the reproductive health questionnaire using computer-assisted personal interviews with trained interviewers. They were asked if they had ‘ever attempted to become pregnant over at least a year without becoming pregnant’, and ‘ever been to a doctor or other medical provider because they have been unable to become pregnant’. Females were classified as having self-reported infertility if they answered “yes” to the first question. Similarly, if they answered affirmatively for the second question, they were classified as having sought reproductive healthcare for infertility. These two types of participants became the main subjects of our study.

### A body shape index (ABSI)

ABSI, the exposure variable, has been recommended as a parameter that manifested the body shape using waist circumference (WC), weight, and height, recently [16]. Data on the WC (cm), weight (kg), and height (cm) of the included individuals were acquired from the 2013–2018 NHANES. WC was measured by placing a flexible tape around the uppermost lateral border of the ilium in a standing condition. Weight and height were measured exactly to one decimal place. Body mass index (BMI) was defined as the weight (kg) divided by the square of the height (m). ABSI value was calculated as a continuous variable using the following equation:

$$\text{ABSI = }\frac{\text{WC}}{{\text{BMI}}^{\text{2/3}}\text{-}{\text{height}}^{\text{1/2}}}$$

### Depression of patient health questionnaire-9 (PHQ-9) score

Depressive symptoms over the past two weeks were assessed by PHQ-9 during face-to-face MEC interviews for all participants. PHQ-9 was widely designed and used as a self-report depression screener for adults in the accompanying editorial [30]. In addition to a depression screener, the PHQ-9 has been proven as a continuous measure and diagnostic method of depression symptom severity. Respondents indicated, on a 0–3 scale, the frequency with which they experienced the following symptoms: (1) anhedonia, (2) depressed mood, (3) sleep disturbance, (4) fatigue, (5) appetite changes, (6) low self-esteem, (7) concentration problems, (8) psychomotor disturbances, and (9) suicidal ideation. The total scores ranged from 0 to 27, whose scores ≥ 10 indicate clinically significant depressive symptoms [23]. Subsequently, depressive symptom severity was divided into 5 levels according to the total score: 0–4, minimal depression; 5–9, mild depression; 10–14, moderate depression; 15–19, moderately severe depression; and 20–27, severe depression [23].

### Covariates

We considered several potential covariates obtained from computer-assisted personal interviews or medical examinations by highly trained medical personnel. Covariates for 5431 participants in this study consisted of sociodemographic variables, lifestyle behaviors, and systematic chronic diseases. Sociodemographic variables included age (continuous variable), race/ethnicity (non-Hispanic white, non-Hispanic black, and others), family income (0-1.30, 1.31–3.50, and ≥ 3.51), and educational background (less than high school, high school or equivalent, and college or above). Recreational activity included none, moderate, and vigorous levels. BMI was utilized as a measure of obesity according to WHO guidelines: (1) normal weight (18.4 kg/m^2^ < BMI ≤ 24.9 kg/m^2^); (2) underweight (BMI ≤ 18.4 kg/m^2^); (3) overweight (24.9 kg/m^2^ < BMI ≤ 30.0 kg/m^2^); (4) and obesity (BMI > 30.0 kg/m^2^). Smoking status (never smoker, former smoker, and current smoker) and hyperlipidemia, and hypertension were also obtained from the interviews.

### Statistical analysis

To accommodate the complex design of the NHANES survey, we incorporated sample weights, clustering, and stratification to generate nationally representative population estimates. The general characteristics of the participants were displayed as weighted mean (standard error (SE)) of continuous variables and counts (weighted percentage (%)) of categorical variables. Weighting variables were used for statistical analysis to increase the representativeness of the population. Distribution differences in sociodemographic and lifestyle behavioral characteristics were first examined across infertility status using an independent t-test or chi-squared test to compare sets of continuous variables and categorical variables, respectively.

Survey-weighted multivariable logistic regression models were established to investigate the associations of infertility (outcome) with ABSI and other individual components (exposure) through odds ratios (OR) and corresponding 95% confidence intervals (CI). The univariable model was not adjusted for confounders, while the multivariable model was adjusted for age, race/ethnicity, educational background, and family income.

**Fig. 2:**
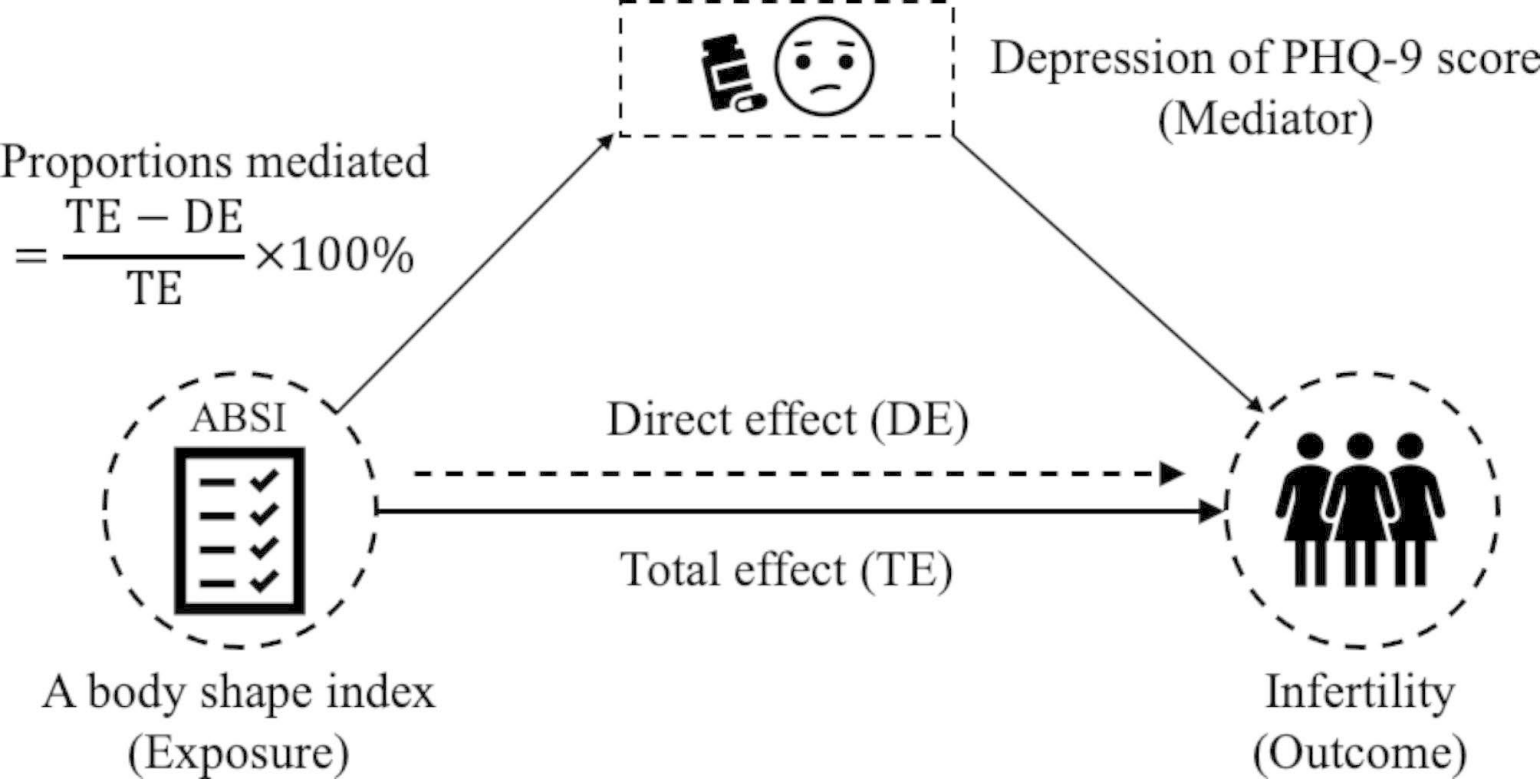
The mediating effect of depression of PHQ-9 score on the relationship between ABSI and infertility among the NHANES population. The diagrammatic drawing illustrated the statistical analysis by setting ABSI as an exposure, depression of PHQ-9 score as a mediator, and infertility as an outcome

A mediation analysis was carried out to examine the mediating role of the PHQ-9 score in the ABSI-infertility among the NHANES population using the R package ‘mediation’ (version 4.5.0). As shown in Fig. 2, ABSI was defined as the exposure, depression of PHQ-9 score as the mediator, and infertility as the outcome. Three steps should be executed in mediation analysis, including the total effect (TE), direct effect (DE), and indirect effect (IE). The TE referred to the sum of the DE and the IE. Typically, the aim of mediation analysis (Fig. 2) was to identify the TE of the exposure (e.g., ABSI) on the outcome (e.g., infertility), the IE of the exposure that acts through a given set of mediators (e.g., PHQ-9 score) of interest and the DE of the exposure unexplained by those same mediators [31]. In our study, the indirect effect of the ABSI on infertility referred to the effect that ABSI acts through PHQ-9 score on the infertility, which could necessarily help us to disentangle the pathways that link ABSI to infertility. Finally, we calculated the mediated proportion by PHQ-9 score by the following formula [32]:

$$\text{Mediated }\text{proportion}\text{ of PHQ-9 score}\text{ = }\frac{\text{total effect-direct effect}}{\text{total effect}}\times 100\%$$

Statistical significance was established at two-sided *p* < 0.05. All analyses in the present study were conducted in R software (version 4.2.0) and relevant packages.

## Results

### Basic information of 5431 NHANES participants

The adult female participants with complete infertility status were our focus of attention. A total of 5431 NHANES participants (38.89 ± 0.27 years old) were ultimately involved in the present study, representing approximately 78.8 million noninstitutionalized residents of the United States. Table 1 presented the characteristics of the NHANES participants by infertility status. Among these, 596 females were diagnosed with infertility, accounting for 10.97% of participants and representing 9.7 million infertility individuals in the United States. Females with infertility were older (42.24 ± 0.55 years old), had higher BMI and ABSI, with higher education and family income levels, and had a higher proportion of current smokers than those without infertility. The infertility group tended to be non-Hispanic white race/ethnicity and diagnose with obesity, hyperlipidemia, and hypertension more than the healthy controls. What’s more, infertility females demonstrated higher PHQ-9 scores and much more severe depressive symptoms, whose recreational activities were less likely to be moderate or vigorous.

**Table 1:** Characteristics of study population in NHANES by infertility status

Characteristics	NHANES (2013–2018)			
	Total	Fertility	Infertility	
Number	5431	4835	596	*p* value
Weighted number	78,815,173	69,028,364	9,786,808	
Continuous variables, weighted mean (SE)				
Age, years	38.89(0.27)	38.42(0.27)	42.24(0.55)	< 0.0001
BMI, kg/m^2^	29.67(0.23)	29.40(0.23)	31.60(0.49)	< 0.0001
PHQ-9 score	3.67(0.10)	3.57(0.10)	4.35(0.26)	0.0077
A body shape index	0.08(0.00)	0.08(0.00)	0.08(0.00)	< 0.0001
Categorical variables, counts (percentage, %)				
Race/ethnicity				
Non-Hispanic white	1808(33.29)	1565(32.37)	243(40.77)	0.0001
Non-Hispanic black	1213(22.33)	1086(22.46)	127(21.31)	
Other	2410(44.38)	2184(45.17)	226(37.92)	
Education background				
Less than high school	321(5.91)	306(6.33)	15(2.52)	< 0.0001
High school or equivalent	1954(35.98)	1775(36.71)	179(30.03)	
College or above	3156(58.11)	2754(59.96)	402(67.45)	
Family income				
0–1.30	1738(32.00)	1578(32.64)	160(26.85)	0.0009
1.31–3.50	1794(33.03)	1591(32.90)	203(34.06)	
≥ 3.51	1415(26.06)	1225(25.34)	190(31.88)	
NA	484(8.91)	441(9.12)	43(7.21)	
Depression measured by PHQ-9				
Minimal, [0,4]	3827(70.47)	3443(71.21)	384(64.43)	0.0010
Mild, [5, 9]	1001(18.43)	880(18.20)	121(20.30)	
Moderate, [10, 14]	362(6.66)	311(6.43)	51(8.56)	
Moderately-Severe, [15, 19]	153(2.82)	124(2.57)	29(4.86)	
Severe, [20, 27]	72(1.33)	63(1.30)	9(1.51)	
NA	16(0.29)	14(0.29)	2(0.34)	
Recreational activity				
None	2664(48.68)	2332(48.23)	312(52.35)	0.0009
Moderate	1367(25.17)	1201(24.84)	166(27.85)	
Vigorous	1420(26.15)	1302(26.93)	118(19.80)	
BMI classification				
Normal, 18.4 < BMI ≤ 24.9	1636(30.12)	1488(30.78)	148(24.83)	< 0.0001
Underweight, BMI ≤ 18.4	123(2.27)	112(2.32)	11(1.85)	
Overweight, 24.9 < BMI ≤ 30.0	1339(24.66)	1223(25.29)	116(19.46)	
Obesity, 30.0 < BMI	2278(41.94)	1965(40.64)	313(52.52)	
NA	55(1.01)	47(0.97)	8(1.34)	
Smoking status				
Never smoker	3790(69.78)	3406(70.45)	384(64.43)	0.0096
Former smoker	674(12.41)	585(12.10)	89(14.93)	
Current smoker	965(17.77)	842(17.41)	123(20.64)	
NA	2(0.04)	2(0.04)	0(0)	
Hyperlipidemia				
No	2210(40.69)	2007(41.51)	203(34.06)	0.0005
Yes	3221(59.31)	2828(58.49)	393(65.94)	
Hypertension				
No	4004(73.72)	3613(74.73)	391(65.60)	< 0.0001
Yes	1427(26.28)	1222(25.27)	205(34.40)	

**Notes**: weighted mean (SE) for continuous variables, and counts (weighted percentage) for categorical variables. P-values were firstly examined across infertility status using an independent t-test or chi-squared test to compare sets of continuous variables and categorical variables, respectively. All p-values were calculated with a two-sided significance level of 0.05. Depression measured by PHQ-9: Minimal, 0 ≤ PHQ-9 ≤ 4; Mild, 5 ≤ PHQ-9 ≤ 9; Moderate, 10 ≤ PHQ-9 ≤ 14; Moderately-Severe, 15 ≤ PHQ-9 ≤ 19; Severe, 20 ≤ PHQ-9 ≤ 27. BMI classification: Normal, 18.4 < BMI ≤ 24.9; Underweight, BMI ≤ 18.4; Overweight, 24.9 < BMI ≤ 30.0; Obesity, 30.0 < BMI.

### Survey-weighted multivariable logistic regression models

As shown in Supplementary Table 1, recreational activity and smoking status have no statistically significance in univariable logistic regression analysis. Therefore, the two variables were not enter into the model. Other variables were subsequently to perform multivariable logistic regression analysis (Table 2). Table 2 demonstrated associations of infertility status with ABSI and other individual components in the NHANES population. Unadjusted OR for ABSI was 0.60 (95% CI: 0.02 to 0.18) in participants diagnosed with infertility. There was a consistent relationship between infertility status and increased ABSI (OR: 0.14, 95% CI: 0.04 to 0.50) in the adjusted model by adjusting age, race/ethnicity, educational background, and family income. Besides, participants with higher PHQ-9 scores were assessed to show higher OR both in the unadjusted model and the adjusted model. In terms of the individual components, infertility was more likely to be associated with obesity and hypertension than in healthy controls. The higher BMI, the higher OR (adjusted OR: 1.03, 95% CI: 1.02 to 1.05) for females with infertility. Moreover, OR was significantly increased in obese individuals in comparison to normal BMI, however, no apparent difference was observed either in the underweight group or in the overweight group. Unadjusted OR for hypertension was 1.73 (95% CI: 1.38 to 2.17), which was slightly attenuated to be 1.54 (95% CI: 1.19 to 1.99) after controlling for the mentioned characteristics. Although the unadjusted OR for hyperlipidemia was 1.34 with statistical significance, there was no significant difference in OR after being modified and optimized in the adjusted model.

**Table 2:** Weighted associations of infertility with ABSI and the individual components in the NHANES population

Characteristics		Unadjusted OR (95% CI)	*p* value	Adjusted OR (95% CI)	*p* value
Continuous variables					
BMI		1.03(1.02,1.04)	< 0.0001	1.03(1.02,1.05)	< 0.0001
PHQ-9 score		1.04(1.01,1.06)	< 0.0001	1.04(1.01,1.07)	< 0.0001
ABSI		0.06(0.02,0.18)	< 0.0001	0.14(0.04,0.50)	< 0.0001
Categorical variables					
BMI classification	Normal	-	-	-	-
	Underweight	1.47(0.53,4.07)	0.4586	1.27(0.36,4.57)	0.7118
	Overweight	0.89(0.63,1.26)	0.5178	0.85(0.57,1.27)	0.4330
	Obesity	1.71(1.30,2.26)	0.0010	1.72(1.23,2.40)	0.0031
Hyperlipidemia	No	-	-	-	-
	Yes	1.34(1.05,1.72)	0.0252	1.13(0.83,1.53)	0.4364
Hypertension	No	-	-	-	-
	Yes	1.73(1.38,2.17)	< 0.0001	1.54(1.19,1.99)	0.0022

**Notes**: Model was adjusted for age, race/ethnicity, educational background, and family income. P-value was calculated through Survey-weighted multivariable logistic regression analysis. All p-values were calculated with a two-sided significance level of 0.05. BMI classification: Normal, 18.4 < BMI ≤ 24.9; Underweight, BMI ≤ 18.4; Overweight, 24.9 < BMI ≤ 30.0; Obesity, 30.0 < BMI.

**Abbreviations**: BMI, body mass index; PHQ-9, patient health questionnaire-9; ABSI, a body shape index; OR, odds ratio; CI: confidence interval

### Mediation analyses

**Fig. 3:**
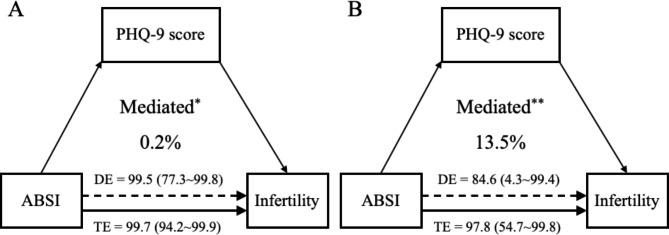
PHQ-9 score played a mediating role in the ABSI-infertility link. **(A)** PHQ-9 score mediated 0.2% of the association between ABSI and infertility in all participants in this study. **(B)** PHQ-9 score mediated 13.5% of the association between ABSI and infertility in overweight and obese participants

In the mediation analyses, the percentage of the mediated effect was calculated by performing a mediation analysis based on the results of TE, DE, and IE. We assessed the percentage mediated through PHQ-9 score for ABSI-infertility in both all participants and partial participants who were grouped into overweight and obesity based on their BMI values (Fig. 3). Among all participants in this study, the TE and DE of ABSI on infertility were 99.7% (95% CI: 94.2–99.9%) and 99.5% (95% CI:77.3–99.8%), respectively. The IE of ABSI on infertility was 0.2% (95% CI: 0.1–16.2%). PHQ-9 score mediated 0.2% (*P* = 0.03) of the association between ABSI and infertility in all participants in this study, however, it mediated 13.5% (*P* < 0.01) in overweight and obese participants. Among the overweight and obese participants, the TE and DE of ABSI on infertility were 97.8% (95% CI: 54.7–99.8%) and 84.6% (95% CI:4.3–99.4%), respectively. The IE of ABSI on infertility was 13.2% (95% CI: 0.3–41.4%).

## Discussion

 ABSI was positively associated with infertility in both overweight and obese female populations, and individuals with high ABSI showed a higher risk of infertility than those with relatively low ABSI. In the mediation analysis, PHQ-9 score was estimated to mediate 0.2% (*P* = 0.03) of the link between ABSI and infertility in all individuals, but to mediate 13.5% (P < 0.01) of the ABSI-infertility association in overweight and obese adult females.

 The close relationship between overweight/obesity and infertility in women was widely acknowledged, which can cause both short-term and long-term adverse consequences for females [33–35]. Previous studies demonstrated that the negative effect obesity brought to fertilization might be due to functional alteration of the hypothalamic-pituitary-ovarian (HPO) axis [35], inflammatory alteration [36], oocyte maturation, and embryo and fetal development [37]. Hence, increasing studies focused on the potential mediators and interventions to reverse the infertile state, represented by weight loss, physical activity, dietary factors, and bariatric surgery [38]. However, the outcomes of these interventions were not satisfactory. It is high time that we figured out a stronger mediator in the ABSI-infertility association

Psychological disorders are becoming more prevalent in modern society. Scientists also have reported women with depressive symptoms tend to suffer from infertility, which could be a result of the altered secretion of sex cycle hormones and endometrial growth affected by emotional disorders [20, 21]. Similarly, the PHQ-9 score and the incidence of psychological depression both substantially increased with the length of infertility [39, 40]. A strong relationship between depression and infertility could be observed. But the relationship between depression score, ABSI, and infertility in overweight and obese females are not clear yet.

 We analyzed the association between ABSI and infertility using NHANES 2013–2018 datasets. Furthermore, the mediating effect of depression score in the association was clarified. As shown in Fig. 3, the mediating effect of the PHQ-9 score in the association of ABSI and infertility in those overweight and obese females (13.5%) is much higher than all participants (0.2%). A previous meta-analysis showed a higher risk of depressive symptoms in obese women [41], and obesity may lead to the alteration of gut microbiota thus influencing the mood and behavior of the host [42]. Behavioral weight control was reported to be an efficient method to improve depressive disorder in obese participants [43]. All these studies were consistent with our findings. Hence, we can conclude that the association between ABSI and infertility seems to be mediated by depression symptoms scored by PHQ-9, especially in those adult females with overweigh and obesity

 Our findings have several clinical implications. First, weight reduction or fat loss has been recognized as a controversial strategy to prevent and treat infertility in overweight or obese women. Some randomized controlled trials have revealed a significantly higher rate of spontaneous conceptions occurring in weight-loss females [44, 45]. Although other studies showed no significant effect of weight loss on fertilization treatment and live birth rate improvement [46, 47]. The contradictory results could be influenced by quantities of factors, such as ethnic groups, sample sizes, and individual heterogeneity. Our present study provided some evidence among USA population during 2013–2018, which is relatively recent data and ethnicity controlled. Second, the mediating effect of the PHQ-9 score in the association of ABSI and infertility in overweight and obese women was demonstrated. Thus, it would be of great importance to evaluate and assess the depression scores and symptoms among those infertile females and promptly provide some clinical help to relieve depressive symptoms. Dadhwal et al. pointed out that family support, sharing problems, and assist with decision-making, and receiving support from spouses could be helpful and beneficial [48].

 We also have some limitations. First, our study was mostly based on the USA population, which may be different from other ethnic populations. So other ethnic groups of overweight and obese females should be further studied. Second, although we took into account confounding factors in our study, residual confounding cannot be eliminated. Although the NHANES database (https://www.cdc.gov/nchs/nhanes/) didn’t directly describe the validity and reliability of questionnaire, information was provided and available on how the questionnaire is used for quality control and monitoring. At least 10% of every interviewer’s work were randomly selected and validated by field supervisors via phone or field visit shortly after the completion of data collection. Questionnaires involved in the present study have been evaluated their validity and reliability in the previous studies [49–52]. Last, we can not conclude a casual relationship with this cross-sectional study. Further clinical trials or genetic assessment between ABSI and infertility in women with overweight and obesity should be complemented.

## Conclusion

 This study pointed out that ABSI is associated with the infertility rate. PHQ-9 score, correlated with depression symptom severity, could be one of the potential mechanisms leading to the positive association between ABSI and infertility in overweight and obese females. It is still necessary and important to conduct further RCTs to confirm whether a lower PHQ-9 score could prevent those overweight and obese women females with high ABSI from infertility. Besides, future clinicians should focus more on the mental health of obese women, which may protect them against infertility.

### Electronic supplementary material

Below is the link to the electronic supplementary material.

Supplementary Material 1

## Data Availability

Data used in this study could be downloaded from https://www.cdc.gov/nchs/nhanes/index.htm. Please contact author for data and material requests (Qiangwei Pan, panqiangwei1984@163.com)
