# Supplementary material for: Depression score mediate the association between a body shape index and infertility in overweight and obesity females, NHANES 2013–2018
Source: BMC Womens Health. 2023 Sep 2;23:471. doi: 10.1186/s12905-023-02622-7 (PMC10475194; doi:10.1186/s12905-023-02622-7)
Supplement: Supplementary file 1 — Supplementary Material 1 [file 12905_2023_2622_MOESM1_ESM.docx]

| Variables | | Unadjusted OR (95% CI) | t | p-value |
| --- | --- | --- | --- | --- |
| Age | | 1.03(1.02,1.03) | 6.95 | <0.0001 |
| BMI | | 1.03(1.02,1.04) | 4.98 | <0.0001 |
| PHQ-9 score | | 1.04(1.01,1.06) | 3.09 | 0.00 |
| ABSI | | 0.06(0.02,0.18) | -5.15 | <0.0001 |
| Race/ethnicity | Non-Hispanic white | - | - | - |
|  | Non-Hispanic Black | 0.81(0.64,1.02) | -1.77 | 0.08 |
|  | other | 0.68(0.52,0.89) | -2.78 | 0.01 |
| Education background | Less than high school | - | - | - |
|  | High school or equivalent | 2.31(1.24,4.32) | 2.64 | 0.01 |
|  | College or above | 3.04(1.63,5.67) | 3.48 | 0.00 |
| Family income | 0–1.30 | - | - | - |
|  | 1.31-3.50 | 1.38(1.07,1.78) | 2.45 | 0.02 |
|  | ≥3.51 | 1.45(1.10,1.91) | 2.66 | 0.01 |
| Depression | Minimal | - | - | - |
|  | Mild | 1.21(0.91,1.62) | 1.3 | 0.20 |
|  | Moderate | 1.44(0.98,2.11) | 1.86 | 0.07 |
|  | Moderately-Severe | 2.21(1.38,3.52) | 3.32 | 0.00 |
|  | Severe | 1.33(0.56,3.14) | 0.65 | 0.52 |
| Recreational activity | None | - | - | - |
|  | Moderate | 0.85(0.64,1.14) | -1.06 | 0.29 |
|  | Vigorous | 0.76(0.56,1.02) | -1.83 | 0.07 |
| BMI classification | Normal | - | - | - |
|  | Underweight | 1.47(0.53,4.07) | 0.75 | 0.46 |
|  | Overweight | 0.89(0.63,1.26) | -0.65 | 0.52 |
|  | Obesity | 1.71(1.30,2.26) | 3.82 | <0.001 |
| Smoking status | Never | - | - | - |
|  | Former | 1.05(0.83,1.33) | 0.4 | 0.69 |
|  | Current | 1.14(0.87,1.50) | 0.95 | 0.35 |
| Hyperlipidemia | No | - | - | - |
|  | Yes | 1.34(1.05,1.72) | 2.32 | 0.03 |
| Hypertension | No | - | - | - |
|  | Yes | 1.73(1.38,2.17) | 4.71 | <0.0001 |

Supplementary table 1. Weighted associations of infertility with ABSI and the individual components in the NHANES population

**Notes:** P-value was calculated through Survey-weighted univariable logistic regression analysis. All p-values were calculated with a two-sided significance level of 0.05. Depression measured by PHQ-9: Minimal, 0≤PHQ-9≤4; Mild, 5≤PHQ-9≤9; Moderate, 10≤PHQ-9≤14; Moderately-Severe, 15≤PHQ-9≤19; Severe, 20≤PHQ-9≤27. BMI classification: Normal, 18.4<BMI≤24.9; Underweight, BMI≤18.4; Overweight, 24.9<BMI≤30.0; Obesity, 30.0<BMI.

**Abbreviations:** BMI, body mass index; PHQ-9, patient health questionnaire-9; ABSI, a body shape index; OR, odds ratio; CI: confidence interval.
